# Recent Advances in the Understanding of Molecular Mechanisms of Resistance in Noctuid Pests

**DOI:** 10.3390/insects12080674

**Published:** 2021-07-27

**Authors:** Gaëlle Le Goff, Ralf Nauen

**Affiliations:** 1 Université Côte d’Azur, INRAE, CNRS, ISA, F-06903 Sophia Antipolis, France; 2 Bayer AG, Crop Science Division, R&D Pest Control, Alfred Nobel Str. 50, 40789 Monheim, Germany

Noctuid moths are among the most devastating crop pests on the planet. Many of them are polyphagous pests capable of feeding on numerous host plants. Their larval stages cause significant damage to plants of agronomic importance, including corn, cotton, soybean, and rice, which result in severe yield losses if not kept under economic damage thresholds. Control of these pest insects is mainly based on the use of chemical insecticides and/or the cultivation of transgenic plants expressing *Bacillus thuringiensis* (Bt) pore-forming insecticidal proteins (Cry toxins). However, the efficacy of these control measures is often jeopardized by the development of resistance due to frequent applications of insecticides and long-term exposure to a relatively small set of Cry toxins expressed in transgenic crops. Understanding the molecular mechanisms of resistance that these pest insects have developed is essential for the implementation of sustainable control methods and resistance management strategies. This special issue is dedicated to this topic and includes 10 papers presenting either original results or reviews.

Several species of the noctuid moth family are invasive species that have recently spread to various parts of the world. The most problematic is probably *Spodoptera frugiperda* (Smith, 1797), which originated from the American continent and recently invaded the Western hemisphere. It was first detected in Africa in 2016 and later in Asia (2018) and Australia (2020). It is approaching Europe and was detected in the Canary Islands (Spain) in July 2020. The Food and Agriculture Organization (FAO) has this year proclaimed it as “one of the most destructive pests jeopardizing food security across vast regions of the globe”. The importance of this species, also known as the fall armyworm (FAW), is reflected in the Special Issue, with half of the publications dedicated to it. Some of the papers focus on the diagnosis to differentiate the two sympatric host plant species of FAW, maize and rice [1], or on the most suitable rearing conditions for this non-model species [2], while others tackle the development of molecular tools for the detection of mutations in insecticide targets conferring resistance [3]. Other studies have monitored the presence of known mutations in the targets of major insecticide classes by comparing native and invasive populations [1,4]. Marked differences exist between FAW populations, which can provide guidance for appropriate resistance management decisions. Indeed, these studies show the presence of mutations in acetylcholinesterase, the target of carbamates and organophosphates, in both native and invasive populations, whereas only native populations show a deletion in ABCC2, the target of some Bt proteins [1,4]. In this case, diamide insecticides could be useful in the fight against FAW since none of the known mutations in the target of diamides, the ryanodine receptor, have been detected. However, it should not be forgotten that resistance can also be acquired through increased capacity to metabolize insecticides and involves detoxification enzymes, such as cytochromes P450 monooxygenases, carboxylesterases, glutathione S-transferases, or transporters like ATP binding cassette (ABC) transporters. Some of the roles of these detoxification genes are highlighted in a review on species in the genus *Spodoptera* [5]. Nevertheless, the fight against insect pests must also include research and discovery of new molecules with different modes of action to counteract resistance mechanisms. Such research should also take into account a societal demand for the development of more environmentally friendly molecules [6].

The other noctuid moth species covered in this Special Issue are also important pests. Klai et al. proposed an analysis of the presence of transposable elements in the genome of *Helicoverpa armigera* and focused on their presence in detoxification genes as they may cause changes in the expression level and confer resistance [7]. Other authors have sought to develop molecular tools either to differentiate between two closely related species, such as *Mythimna loreyi* and *Mythimna separata*, with the LAMP (loop-mediated isothermal amplification) assay [8], or by designing primers to monitor the expression of Bt toxin target genes and reference genes in soybean looper, *Chrysodeixis includens* [9].

ABC transporters are important Bt protein receptors and there is an increasing body of evidence that mutations in ABC transporters (e.g., ABCC2) confer high levels of resistance to Cry toxins as reviewed in [10]. In his review, Heckel [10] highlights a number of research questions that remain open, such as how potential Cry toxin receptor proteins and Cry toxins interact to form pores. However, our knowledge and understanding regarding the function and regulation of genes conferring resistance in pests is constantly increasing and particularly facilitated by the exploitation of new technologies, such as genome editing by CRISPR/Cas9.

Finally, we would like to thank all authors for their commitment to contribute their articles and the many reviewers for their valuable comments and suggestions helping all of us to shape this interesting Special Issue, which hopefully attracts a broad readership of *Insects*.

## Data Availability

Not applicable.
